# Effect of local infiltration analgesia, peripheral nerve blocks, general and spinal anesthesia on early functional recovery and pain control in unicompartmental knee arthroplasty

**DOI:** 10.1186/s12891-018-2165-9

**Published:** 2018-07-24

**Authors:** M. T. Berninger, J. Friederichs, W. Leidinger, P. Augat, V. Bühren, C. Fulghum, W. Reng

**Affiliations:** 1 endogap, Joint Replacement Institute, Garmisch-Partenkirchen Medical Center, Auenstr. 6, 82467 Garmisch-Partenkirchen, Germany; 2 Department of Trauma Surgery, BG Trauma Center Murnau, Prof.-Küntscher Str. 8, 82418 Murnau, Germany; 3 Department of Anesthesiology and Intensive Care, Garmisch-Partenkirchen Medical Center, Auenstr. 6, 82467 Garmisch-Partenkirchen, Germany; 4 Institute of Biomechanics, BG Trauma Center Murnau, Prof.-Küntscher Str. 8, 82418 Murnau, Germany; 5 Institute of Biomechanics, Paracelsus Medical University, Strubergasse 21, 5020 Salzburg, Austria

**Keywords:** Local infiltration analgesia, Femoral nerve block, Unicompartmental knee arthroplasty, Epidural catheter, General anesthesia, Spinal anesthesia

## Abstract

**Background:**

The aim of the study was to analyze the effect of local infiltration analgesia (LIA), peripheral nerve blocks, general and spinal anesthesia on early functional recovery and pain control in primary unicompartmental knee arthroplasty (UKA).

**Methods:**

Between January 2016 until August 2016, 134 patients underwent primary UKA and were subdivided into four groups according to their concomitant pain and anesthetic procedure with catheter-based techniques of femoral and sciatic nerve block (group GA&FNB, *n* = 38) or epidural catheter (group SP&EPI, *n* = 20) in combination with general anesthesia or spinal anesthesia, respectively, and LIA combined with general anesthesia (group GA&LIA, *n* = 46) or spinal anesthesia (group SP&LIA, *n* = 30). Outcome parameters focused on the evaluation of pain (NRS scores), mobilization, muscle strength and range of motion up to 7 days postoperatively. The cumulative consumption of (rescue) pain medication was analyzed.

**Results:**

The LIA groups revealed significantly lower (about 50%) mean NRS scores (at rest) compared to the catheter-based groups at the day of surgery. In the early postoperative period, the dose of hydromorphone as rescue pain medication was significantly lower (up to 68%) in patients with SP&EPI compared to all other groups. No significant differences could be detected with regard to grade of mobilization, muscle strength and range of motion. However, there seemed to be a trend towards improved mobilization and muscle strength with general anesthesia and LIA, whereof general anesthesia generally tended to ameliorate mobilization.

**Conclusions:**

Except for a significant lower NRS score at rest in the LIA groups at day of surgery, pain relief was comparable in all groups without clinically relevant differences, while the use of opioids was significantly lower in patients with SP&EPI. A clear clinically relevant benefit for LIA in UKA cannot be stated. However, LIA offers a safe and effective treatment option comparable to the well-established conventional procedures.

## Background

Anteromedial knee osteoarthritis is a distinct clinicopathological entity which often leads to disabling pain and limitation of range of motion [1]. If conservative treatment fails, unicompartmental knee arthroplasty (UKA) is a good treatment option achieving good-to-excellent results and a 10-year survivorship up to 96% [2]. Compared with total knee arthroplasty (TKA), benefits of UKA include joint and bone preservation by replacing only one compartment and preservation of the other intact weight-bearing compartment as well as the anterior and posterior cruciate ligaments. This contributes to reducing intraoperative blood loss and postoperative pain for early rehabilitation. The unicompartmental implants are less bulky and allow for almost normal knee kinematics. All in all, UKA allows patients a faster return to a more functional level than TKA [3–5]. However, although unicompartmental arthroplasty with a minimally invasive technique results in less operative trauma, moderate to severe pain postoperatively remains a common problem, which restricts early mobilization and functional recovery.

Along with UKA, minimally invasive surgery became more and more popular in the early 1990s [6]. Besides a more soft tissue sparing surgical approach, minimally invasive surgery includes a faster recovery with a greater range of motion and less peri- and postoperative pain. This, however, also requires improvements in the postoperative care of physical therapy, the anesthetic techniques and postoperative pain management being significant contributors to an accelerated recovery and pain-free result. This led to the introduction of a perioperative multimodal approach to pain management including modified analgesic techniques of peripheral and epidural nerve blocks and local intraarticular injections [7]. Catheter-based techniques as femoral nerve blocks (FNB) usually result in a sufficient pain relief [8]. However, the catheter themselves limit the patients´ ability to ambulate in the immediate post-operative period until the catheters are removed after some days. Furthermore, the motor impairment, quadriceps weakness, and risk of nerve injury can lead to a longer usage of knee immobilizer or crutches to avoid falls in the intermediate postoperative period [9, 10].

As an alternative method for multimodal, postoperative pain management, local infiltration analgesia (LIA) around the soft tissues of the knee joint gained increasing interest in recent years with excellent pain relief and absent muscle weakness [11]. This led to many studies comparing LIA with peripheral nerve blocks showing comparable results with regard to the postoperative pain [12–14] with slight advantages of the LIA technique in the early postoperative period [15, 16]. Data comparing LIA with continuous epidural analgesia are limited and favor LIA over continuous epidural analgesia [17–19]. However, all these studies have in common, that they are performed with patients who have received a TKA.

In literature, there are only a few trials, which described the LIA technique in UKA. In preliminary trials, Beard et al. [20] and Reilley et al. [21] tested the LIA technique in unicompartmental arthroplasty and presented promising results in terms of patient satisfaction and pain relief. Essving et al. showed a significantly shorter median hospital stay, lower postoperative pain (at rest and with movement) within the first day and lower morphine consumption in the LIA group compared to a control group which received saline instead [22]. However, the LIA mixture or saline was injected both intraoperatively and 21 h postoperatively via an intraarticular catheter. Therefore, an interpretation of the results is difficult.

In literature, there is no study analyzing the effect of LIA, (peripheral) catheter-based techniques and their combination with general or spinal anesthesia on pain control, mobilization, muscle strength and range of motion for up to 7 days postoperatively in one patient collective of UKA. Therefore, the aim of the study was to analyze the effect of these different peri- and postoperative anesthetic therapies on early functional recovery and pain control in primary UKA.

## Methods

### Patients

One hundred thirty four patients were treated for medial knee osteoarthritis with UKA between January and August 2016 and were included for this retrospective analysis. The inclusion criterion was primary medial knee osteoarthritis. Patients were excluded if they had significant patellofemoral or lateral osteoarthritis, secondary arthritis due to rheumatoid arthritis or trauma, osteonecrosis or revision surgery. Patient demographics and clinical data are shown in Table 1. Depending on the anesthetic procedure and the peri−/postoperative pain management, patients were divided into 4 groups as follows: 38 patients received general anesthesia in combination with a FNB and sciatic nerve block (GA&FNB), 20 patients spinal anesthesia combined with epidural anesthesia (SP&EPI), 46 patients general anesthesia and LIA (GA&LIA) and 30 patients spinal anesthesia and LIA (SP&LIA).

**Table 1:** Patients demographics and clinical data

	GA&FNB	SP&EPI	GA&LIA	SP&LIA
Patients	*n* = 38	*n* = 20	*n* = 46	*n* = 30
Gender	f = 20; m = 18	f = 12; m = 8	f = 23; m = 23	f = 10; m = 20
Age	68 ± 9.0	70 ± 9.2	68 ± 9.4	69 ± 8.4
Piritramide	OP: *n* = 14 (*36.8%*): 10.7 mg ± 5.8	OP: n = 2 (*10%*): 8.3 mg ± 1.1	OP: *n* = 29 (*63.0%*): 8.1 mg ± 4.5	OP: n = 3 (*10.0%*): 7.5 mg ± 0.0
	Day 1: n = 4 (*10.5%*): 9.4 mg ± 3.8	Day 1: -	Day 1: n = 2 (*4.4%*): 7.5 mg ± 0.0	Day 1: n = 4 (*13.3%*): 9.4 mg ± 3.8
	Day 2: n = 3 (*7.9%*): 7.5 mg ± 0.0	Day 2: -	Day 2: -	Day 2: -
Implant fixation	cementless: *n* = 20 (*52.6%*)	cementless: *n* = 11 (*55.0%*)	cementless: *n* = 23 (*50.0%*)	cementless: *n* = 16 (*53.3%*)
	hybrid *n* = 10 (*26.3%*)	hybrid *n* = 5 (*25.0%*)	hybrid *n* = 9 (*19.6%*)	hybrid n = 5 (*16.7%*)
	cemented *n* = 8 (*21.1%*)	cemented n = 4 (*20.0%*)	cemented n = 14 (*30.4%*)	cemented n = 9 (*30.0%*)
Salvage pain management	n = 1 (2.6%)	n = 1 (2.6%)	n = 1 (2.6%)	–
	→PCIA: n = 1	→PCIA: n = 1 →3in1: *n* = 1 →G.A.: n = 1	→3in1: n = 3 →PCIA: n = 1	
LIA	–	–	100 ml	100 ml
Dexamethasone	–	–	15.4 mg ± 3.1	16.6 mg ± 2.1

This study was performed in conformity with the Declaration of Helsinki and was approved by the Ethics Committee of the Bavarian State Chamber of Physicians (ID: 2017–109).

### Anesthetic techniques

After induction of general anesthesia, patients allocated to group GA&FNB had a FNB catheter inserted with real-time monitored ultrasound imaging. A total of 20 ml of 0.1% ropivacaine was injected around the femoral nerve; additionally ultrasound-guided sciatic nerve block with 20 ml of 0.1% ropivacaine was established as single shot block. Postoperatively, 0.2% ropivacaine was continuously infused at the rate of 3 ml/h for 3 days through the femoral catheter.

In group SP&EPI, a catheter was preoperatively sited at the cranial lumbar vertebrae.

combined with a spinal anesthesia (1 ml of 0.5% bupivacaine and 10 μg sufentanil in the subarachnoid space) in a single needle technique. After recovery from spinal anesthesia under the level L3, an initial 10 ml bolus containing 0.5% bupivacaine, 0.6 μg/ml sufentanil and saline was introduced. Thereafter, patients were self-medicated with a bolus of 4 ml via a patient-controlled epidural anesthesia (PCEA) system with a lockout of 20 min. PCEA was discontinued three days after surgery.

### Local infiltration analgesia (LIA)

In the LIA groups, 100 ml of 0.2% ropivacaine without any additional components were intraoperatively administered by the surgeon periarticularly in the soft tissues according to the injection technique popularized by Kerr and Kohan [11]. During the beginning of the narcosis, 0,2 mg/kg dexamethasone was injected intravenously. All infiltration was done using 25-ml syringes and 10-cm-long 19-G spinal needles. The LIA solution was administered after completion of all femoral and tibial osteotomy steps, immediately before cement fixation of the tibial component. The LIA solution was systematically injected into the tissues around the knee joint according to a standardized protocol: in the medial and lateral tibial and femoral periosteum as well as medial and lateral posterior articular capsule, and in the subcutaneous tissue, in the Hoffa fat pad and finally intraarticularly after capsular suture.

### Surgery

All surgeries were performed by three senior surgeons. Intra-operatively, single-shot cefazolin 2 g (or clindamycin 600 mg in case of incompatibility of penicillin) for infection prophylaxis was given to all patients. The surgeries were performed with a standard minimal invasive midline vertical incision and medial parapatellar approach; the patella was removed laterally but not dislocated or everted. A tourniquet was inflated to 250 mmHg at the beginning of the surgery and deflated after removal of the surgical dressings. In all cases, the Oxford**®** Partial Knee System (Zimmer Biomet, Warsaw, IN, USA) was used. Implants were fixated cementless, hybrid cemented (cemented tibial and cementless femoral component) or fully cemented depending on bone stock and age (see Table 1). Bone resections and implant insertion were performed according to the manufacturers manual.

### Postoperative pain management and care

Postoperative management was identical in all groups. After surgery, every patient was given a peripheral pain medicament (WHO grade I, e.g. paracetamol, metamizole, ibuprofen or diclofenac) for about 2 weeks to relieve pain and low molecular weight heparins subcutaneously for about 2 weeks to prevent deep vein thrombosis. The cumulative doses of rescue analgesia (hydromorphone p.o. or piritramide i.v.) were also registered.

Postoperative physiotherapy was started immediately after surgery in a progressive manner and all patients received physiotherapy daily. A specially trained pain service regularly visited all patients twice a day for the first four postoperative days.

### Outcome measures

Self-reported pain scores in terms of numeric rating scores (NRS) at rest and with activity (0 = no pain; 10 = worst pain) from day of surgery until postoperative day 4 were collected and analyzed. For evaluation of functional outcomes, grade of mobilization ranging from values of 1 to 6 according to our institutional grading system of mobilization was analyzed: 1 = bedridden, 2 = sitting, 3 = standing, 4 = walking in room, 5 = walking on the floor, 6 = walking stairs. Furthermore, muscle strength according to the British medical research council (M0/5-M5/5) and passive range of motion (degrees of extension and flexion) were examined. Functional outcomes of mobilization, muscle strength and range of motion were documented daily from pre-operative day until postoperative day 7, respectively. The patients’ medical files were also studied for potential analgesic technique-related and surgery-related complications within the first 7 days, such as rates of neurologic events, cardiovascular events, falls, knee joint infections, prosthesis loosening, or revision surgery. All data were collected from the patients´ medical records and nurses´ observational charts.

### Data analysis

Statistical analysis was performed with SPSS statistical software 20.0 (SPSS for Windows, ver. 20.0; SPSS, Chicago, IL, USA). Descriptive statistics were calculated for all variables of interest. Continuous measures such as age were summarized using means and standard deviations whereas categorical measures were summarized using counts and percentages.

The Kruskal-Wallis test was used for analysis of one nominal variable and one ranked variable. In a further detailed analysis, post-hoc comparisons of factor-level combinations were conducted by use of Mann-Whitney-U test, depending on previous (overall) significance testing. In this explorative study, no adjustment of the alpha-error level was conducted.

## Results

Baseline characteristics of patients were comparable among all groups (Table 1). No patient suffered from chronic pain in daily life with use of opioids prior to the surgery. Approximately 50% of the implants were fixated cementless while the other 50% included hybrid cemented (cemented tibial and cementless femoral component) or fully cemented implant fixation.

Pain exacerbation after surgery due to insufficient pain relief (NRS > 7) with the current anesthetic technique led to another analgesic technique. In GA&FNB, one patient received a patient-controlled intravenous analgesia (PCIA) with an initial bolus of 4 mg piritramide followed by an optional bolus of 2 mg piritramide with a lockout of 10 min. In SP&EPI, 3 patients (15.0%) were converted to PCIA (*n* = 1), secondary application of a FNB (n = 1) and to general anesthesia (*n* = 1). In GA&LIA, 4 patients (8.7%) were changed to FNB (*n* = 3) and PCIA (n = 1). These patients were excluded from analysis. In SP&LIA, a modification of anesthetic regime was not necessary for any patient. No analgesic technique-related and surgery-related complication was encountered in any group within the first postoperative 7 days. At the day of surgery, the demand for piritramide was significantly higher (51% vs. 10%; *p* < 0.05) in groups with general anesthesia compared to spinal anesthesia. All LIA patients received 100 ml of the LIA mixture with 15.4 mg ± 3.1 (GA&LIA) and 16.6 mg ± 2.1 (SP&LIA) dexamethasone, respectively.

### Pain

**Fig. 1:**
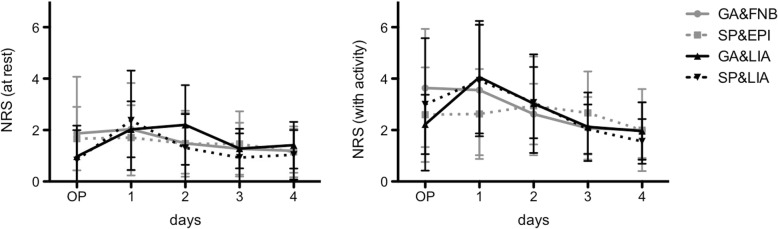
Numeric Rating Scores at rest (left) and with activity (right) are presented for day of surgery (OP) and postoperative days 1 to 4

At the day of surgery, the NRS scores at rest of the LIA groups were statistically significant lower (GA&LIA: 1.0 ± 1.0; SP&LIA: 0.8 ± 1.3) compared to the catheter-based groups (GA&FNB: 1.9 ± 2.2; SP&EPI: 1.7 ± 1.2; *p* < 0.05) (Fig. 1). At any further time point, the NRS scores did not show any significant differences (*p* > 0.05). The values of the LIA groups slightly increased at day 1 while the catheter-based groups showed almost constant pain values at the day of surgery and day 1. Afterwards, a gradual reduction of pain values was detectable.

The development of the NRS scores with activity was comparable among groups (p > 0.05) (Fig. 1). In comparison with the NRS scores at rest, SP&EPI showed an almost constant pain score during these days. The NRS value of GA&LIA was the lowest of all groups at the day of surgery (2.2 ± 1.2); however, the pain nearly doubled at postoperative day 1 (4.1 ± 2.2) to diminish again at day 2 (3.0 ± 1.9), which was similar in SP&LIA (day 1: 3.9 ± 2.2; day 2: 3.1 ± 1.4).

**Fig. 2:**
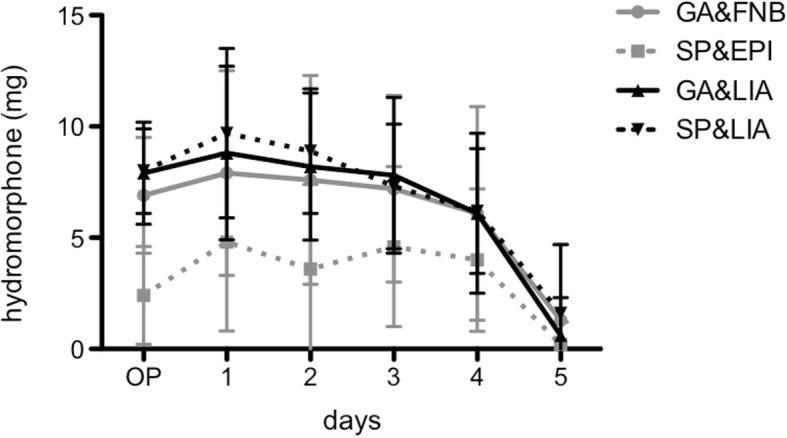
The cumulative dose of hydromorphone (in mg) for all groups at day of surgery until postoperative days 4 is shown

At the day of surgery as well as at postoperative days 1, 2 and 3, the doses of hydromorphone were on average 38 to 68% lower in SP&EPI compared to all other groups (*p* < 0.05) (Fig. 2). The dose of hydromorphone seemed to slightly increase in all groups on postoperative day 1 in order to gradually fall afterwards.

### Mobilization

**Fig. 3:**
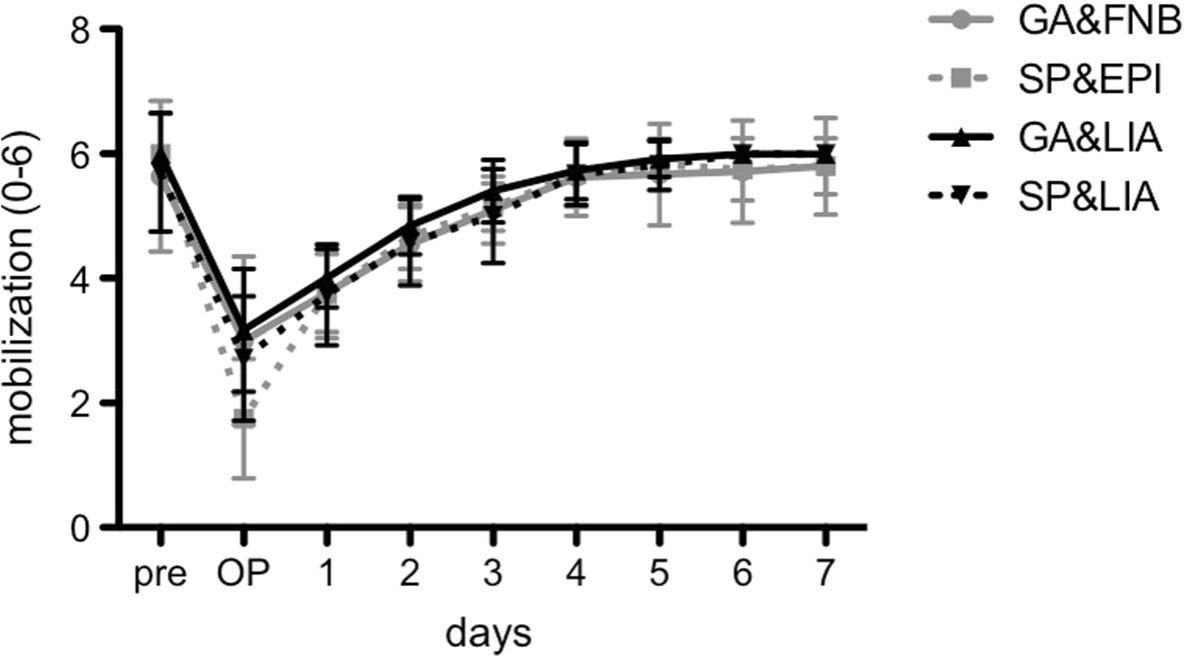
The grade of mobilization (0 to 6) revealed a gradual increase after surgery until postoperative day 7

Upon analyzing the grade of mobilization, no significant differences among the groups at any time point were observed (Fig. 3). All patients with general anesthesia were able to stand up (values≥3) at the day of surgery (GA&FNB: 3.0 ± 1.3; GA&LIA: 3.2 ± 1.0) while patients with spinal anesthesia showed non-significantly (*p* = 0.121) lower values (SP&EPI: 1.8 ± 1.0; SP&LIA: 2.7 ± 1.0) and reached full standing as recently as on postoperative day 1. From day 2 on, all patients were able to walk in the room or even on the floor (values≥4) and the slight differences in mobilization among the groups were diminished. Overall, GA&LIA still tended to achieve a slightly accelerated and earlier mobilization compared to all other groups, particularly at the day of surgery with on average 21% higher grade of mobilization while at later time points this trend diminished to about 6% (*p* > 0.05).

### Muscle strength

**Fig. 4:**
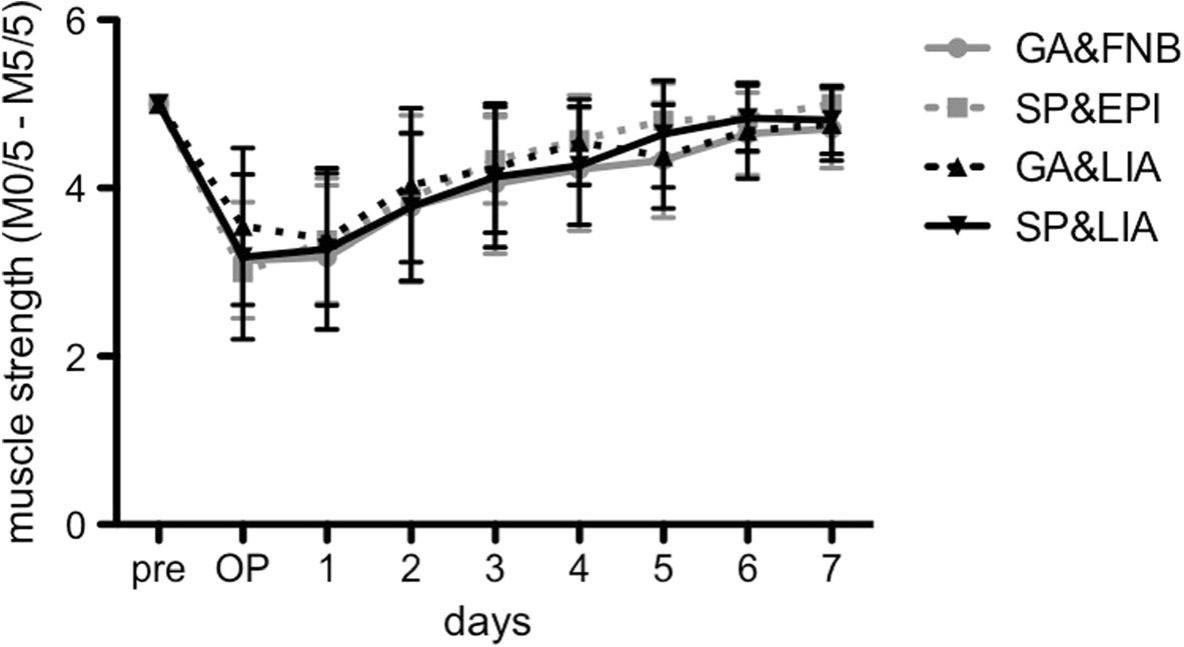
The grade of muscle strength according to the British medical research council (M0/5-M5/5) after surgery until postoperative day 7 is shown

There were no significant differences (*p* > 0.05) among the groups with respect to muscle strength (M0/5-M5/5) (Fig. 4). At the day of surgery, all patients showed reduced strength and the knee joint could only be moved without examiner’s resistance (GA&FNB: 3.1 ± 0.7; SP&EPI: 3.0 ± 0; GA&LIA: 3.6 ± 0.9; SP&LIA: 3.2 ± 1.0). From day 3 on, muscle strength increased daily and the knee joint could be moved against resistance (values> 4 in all groups). GA&LIA seemed to show ameliorated muscle strength in the early postoperative period. Afterwards, the spinal anesthesia groups revealed comparably higher values (*p* > 0.05).

### Range of motion

**Fig. 5:**
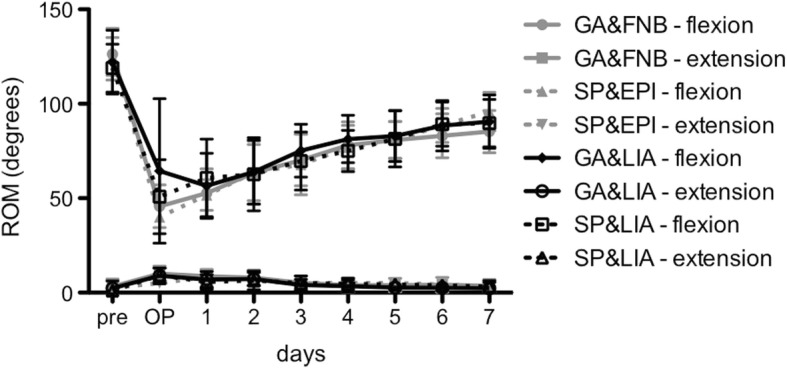
Degrees of range of motion (flexion and extension) of all groups from preoperative day to postoperative day 7 are presented

The mean range of motion (flexion and extension of the knee joint) was similar within the groups (Fig. 5). At the day of surgery, the LIA groups showed a non-significantly improved flexion (GA&LIA: 64.4° ± 38.2° and SP&LIA: 50.8° ± 19.6°) compared to the catheter-based groups (GA/FNB: 45.7° ± 11.3° and SP&EPI: 40.0° ± 0°). Afterwards, these slight differences diminished and the flexion gradually increased while extension decreased. Considering all groups, all patients reached 80° of flexion at day 5.

## Discussion

In recent years, several studies described the benefit of LIA as an alternative analgesic procedure in TKA [15, 16, 23, 24]; studies in UKA are rare [20–22]. While Beard et al. [20] and Reilley et al. [21] revealed encouraging results of patient satisfaction and pain relief, Essving et al. particularly showed lower postoperative pain at rest and with movement within the first day and lower morphine consumption in the LIA group [22]. The authors compared the results to a control group, which received saline. However, both were injected intraoperatively and, additionally, 21 h postoperatively via an intraarticular catheter.

At the day of surgery, the LIA technique revealed about 50% lower mean NRS values at rest (0.9 ± 0.1) compared to the catheter-based groups (1.8 ± 0.1). However, even when being statistically significant these findings are not clinically relevant because the differences are too marginal to reach a clinical impact. At postoperative day 1, pain values of NRS scores with activity in LIA with general or spinal anesthesia, respectively, nearly doubled to decrease again at day 2 indicating a pain exacerbation at day 1. A slight increase of the dose of hydromorphone was also visible at day 1. However, the results of the significant differences in the dose of hydromorphone in patients with SP&EPI within the early postoperative days may be very well of clinical relevance; alone by the not negligible side effects including nausea, dizziness and constipation [25], which can restrict early mobilization, particularly in elderly patients. In the early postoperative period, the dose of hydromorphone was the lowest (up to 68%) in SP&EPI compared to all others. This is a clear advantage of this analgesic treatment in UKA in our patient collective. In a study analyzing fast-track recovery by use of general and spinal anesthesia, Munk et al. described a high level of pain during the first postoperative night and the next day [26]. At the day after surgery, patients needed 10 mg opioid (oxycodone), which was comparable with our result with mean hydromorphone of 9.3 mg at postoperative day 1 in the LIA groups. It is questionable if patients with this high need of opioid usage should be discharged at day after surgery as performed in the study of Munk. The authors, however, also stated that due to this high level of pain and use of strong opioids in the initial period after surgery, there is no recommendation for UKA in an outpatient procedure unless perioperative analgesia is improved. The use of opioids need a strict surveillance and we therefore strongly do not support a discharge before opioids have been decreased significantly.

Interestingly, we did not find any significant differences in terms of mobilization, muscle strength or range of motion. It is well known, that UKA resulted in improved and faster postoperative knee function compared to TKA [3–5]. The effect of changing perioperative analgesic strategies in UKA might be too small to further improve these functional outcome parameters. Our results only showed a trend towards improved mobilization and muscle strength with the combination of general anesthesia and LIA, whereof general anesthesia generally tended to ameliorate mobilization. An advantage of general anesthesia in terms of functional parameters compared to spinal anesthesia is not surprising since the continuous epidural infiltration of anesthetics for three days, of course, affects muscle strength and thereby, decreases function.

Finally, it is up to the treating surgeon to choose a procedure, which is individually and thoroughly adapted to the patient. It is important to obtain a detailed medical history and clinical examination upon meeting the patient including analysis of the individual sense of pain and knee function. It has to be considered individually whether the advantage of spinal anesthesia and epidural catheter in terms of comparably lower dose of opioids predominantly exceeds the slightly poorer function. Patients with good preoperative knee function and muscle strength may physically and mentally benefit from decreased pain postoperatively by use of spinal anesthesia; while patients who predominantly suffer from functional restrictions rather than chronic knee pain might be more satisfied with LIA.

Originally, Kerr and Kohan described the LIA mixture as a combination of ropivacaine 2 mg/ml, ketorolac 30 mg and adrenaline 10 mg/ml that is infiltrated in different layers of the joint in volumes of 150–170 ml for TKA [11]. However, in subsequent years different mixtures, containing among others opioids and steroids, non-steroidal anti-inflammatory drugs, morphine, and epinephrine have been used in addition to local anesthetics without reaching a consensus on a certain dose or drug combination [27–31]. As local anesthetics both bupivacaine and ropivacaine are regularly used for LIA in clinic. Thereby, ropivacaine offers a long-acting profile with reduced cardiotoxicity compared to bupivacaine and intrinsic vasoconstrictor properties [32].

In the present study, we only used 100 ml of 0.2% ropivacaine for infiltration for several reasons. In knee arthroplasty, chondrolysis due to injected local anesthetics is mainly of concern after UKA, since there is still healthy cartilage remaining in the joint. With numerous studies demonstrating chondrotoxicity of local anesthetics in human and animal joints, it is very important to understand the molecular mechanisms and clinical effects of these medications on chondrocytes including decreased cell metabolism, increased apoptosis, necrosis and morphologic tissue degeneration and thereby, the risk for early osteoarthritis [33–36].

The chondrotoxic effects occur dose- and time-dependent [37–39]. In literature, there is no study that could show a significant chondrotoxic effect with low concentrations of bupivacaine (0.0625%) or ropivacaine (0.1 and 0.2%) [33]. Higher concentrations, however, led to a significant chondrocyte cell death [40–42]. Piper et al. compared the in vitro toxicity of bupivacaine and ropivacaine in human articular chondrocytes and showed that 0.5% ropivacaine is significantly less toxic than 0.5% bupivacaine in both intact human articular cartilage and chondrocyte culture [35]. In the study of Grishko et al., exposure of primary human chondrocytes to single-dose ropivacaine (0.5 and 0.2%) did not affect chondrocyte viability after 24 h [41]. However, after 5 days, a significant decrease of viable cells at all concentrations of lidocaine, bupivacaine, and ropivacaine analyzed, were detected, except for 0.2% ropivacaine. In UKA, Essving et al. also did not see any clinical evidence of chondrolysis during the 6 months of follow up after LIA with high-dose ropivacaine (400 mg) [43]. In terms of clinical use, we conclude in accordance with the current literature that ropivacaine at very low concentrations should be preferably used over bupivacaine [33].

Furthermore, a direct linear relationship of increasing cell death with increasing duration of exposure has been described in prior studies [38, 44, 45]. Long-term exposure is promoted by continuous intraarticular application of local anesthetics. Several studies described the continuous LIA in order to prolong its effect by use of e.g. infusion pumps [46, 47]. The results are varying: In a randomized double-blind study, Ali et al. did not show any clinically relevant effect on VAS pain, analgesic consumption, range of motion or length of hospital stay with continuous intraarticular analgesia after TKA [48]. However, a higher risk of wound-healing complications including deep infections was described [48, 49]. In another study comparing single-injection and continuous LIA, continuous infiltration resulted in prolonged superior analgesia and was associated with better functional recovery and patient satisfaction [50]. In terms of accelerated chondrotoxicity and risk for infection after prolonged exposure, we recommend avoiding using continuous infiltration or even additional single shots after some hours.

There are some limitations that pertain to that study. Due to its retrospective design, the study was not blinded or randomized, which may have introduced reporting bias. Furthermore, the choice of anesthesia by the patient might have induced some selection bias, although the group characteristics appeared to be identical among the four groups. Although it was a retrospective investigation, the strengths of the study include a large number of patients managed according to clear inclusion and exclusion criteria. The surgical and anesthetic procedures followed a consistent standard-treatment protocol in the same hospital by the same surgeons with extensive surgical experience in the treatment of UKA and its concomitant analgesic procedures.

## Conclusions

In conclusion, the findings from this study suggest a slight but clinically not relevant advantage of the LIA groups in the early postoperative period in terms of mobilization, muscle strength and range of motion. In general, pain relief was similar in all groups, with exception of a significant lower NRS score at rest in the LIA groups at day of surgery. The use of rescue pain medication was significantly lower in patients with SP&EPI. A clear clinically relevant benefit for LIA in UKA can not be stated. Preoperative information including knee function and pain status should be considered for each patient individually before choosing a multimodal perioperative analgesia protocol. In UKA, infiltration of a local anesthetic offers a safe and effective treatment option comparable to the well-established conventional procedures.

## Abbreviations

FNB: femoral nerve block
GA&FNB: General anesthesia and femoral nerve block (+sciatic nerve block)
GA&LIA: General anesthesia and local infiltration analgesia
LIA: local infiltration analgesia
NRS: Numeric rating scale
OP: Operation (day of surgery)
PCIA: Patient-controlled intravenous analgesia
SNB: Sciatic nerve block
SP&EPI: Spinal anesthesia and epidural catheter
SP&LIA: Spinal anesthesia and local infiltration analgesia
TKA: Total knee arthroplasty
UKA: Unicompartmental knee arthroplasty
